# Case-case-control study on factors associated with *vanB* vancomycin-resistant and vancomycin-susceptible enterococcal bacteraemia

**DOI:** 10.1186/1471-2334-14-353

**Published:** 2014-06-28

**Authors:** Agnes Loo Yee Cheah, Trisha Peel, Benjamin P Howden, Denis Spelman, M Lindsay Grayson, Roger L Nation, David CM Kong

**Affiliations:** 1 Centre for Medicine Use and Safety, Monash University, 381 Royal Parade, Parkville, Victoria, Australia; 2 Department of Infectious Diseases, The Alfred, Melbourne, Victoria, Australia; 3 Infectious Diseases Department, Austin Health, 145 Studley Road, Heidelberg, Victoria, Australia; 4 Department of Surgery, St Vincent’s Health, University of Melbourne, Melbourne, Australia; 5 Department of Medicine, University of Melbourne, Melbourne, Australia; 6 Microbiology Department, Austin Health, 145 Studley Road, Heidelberg, Victoria, Australia; 7 Department of Microbiology and Immunology, University of Melbourne, Melbourne, Australia; 8 Microbiology Unit, The Alfred, Melbourne, Victoria, Australia; 9 Monash University, Victoria, Australia

**Keywords:** Enterococci, Vancomycin-resistant, Vancomycin-susceptible, Bacteraemia

## Abstract

**Background:**

Enterococci are a major cause of healthcare-associated infection. In Australia, *vanB* vancomycin-resistant enterococci (VRE) is the predominant genotype. There are limited data on the factors linked to *vanB* VRE bacteraemia. This study aimed to identify factors associated with *vanB* VRE bacteraemia, and compare them with those for vancomycin-susceptible enterococci (VSE) bacteraemia.

**Methods:**

A case-case-control study was performed in two tertiary public hospitals in Victoria, Australia. VRE and VSE bacteraemia cases were compared with controls without evidence of enterococcal bacteraemia, but may have had infections due to other pathogens.

**Results:**

All VRE isolates had *vanB* genotype. Factors associated with *vanB* VRE bacteraemia were urinary catheter use within the last 30 days (OR 2.86, 95% CI 1.09-7.53), an increase in duration of metronidazole therapy (OR 1.65, 95% CI 1.17-2.33), and a higher Chronic Disease Score specific for VRE (OR 1.70, 95% CI 1.05-2.77). Factors linked to VSE bacteraemia were a history of gastrointestinal disease (OR 2.29, 95% CI 1.05-4.99) and an increase in duration of metronidazole therapy (OR 1.23, 95% CI 1.02-1.48). Admission into the haematology/oncology unit was associated with lower odds of VSE bacteraemia (OR 0.08, 95% CI 0.01-0.74).

**Conclusions:**

This is the largest case-case-control study involving *vanB* VRE bacteraemia. Factors associated with the development of *vanB* VRE bacteraemia were different to those of VSE bacteraemia.

## Background

Globally, enterococci are a major cause of nosocomial infection [1-3]. Of concern is vancomycin-resistant enterococci (VRE) bacteraemia, which has been associated with higher mortality and morbidity compared to VRE wound, urinary tract and intra-abdominal infections [4]. In Australia, *vanB* is the major genotype [3,5], in contrast to the United States (US) where *vanA* is predominant [2,6]. Importantly, VRE is increasingly isolated in clinical settings [3], and has emerged in the community viz. aged-care facilities and outpatient clinics [7,8].

Studies which examined the factors associated with VRE colonisation and/or a range of different VRE infections have either had *vanA* genotype as the predominant strain (as reported or through personal communication with the authors) [9-14], or did not report the genotype [4,15-29]. All aforementioned studies were performed in the US, where *vanA* is predominant [2]. Only two studies examining factors linked to the development of VRE bacteraemia involved predominantly the *vanB* genotype [30,31]. The clinical profile of a colonised versus an infected patient is different [32]. Thus, studies [4,23,25-27] that have included both colonised and infected patients as their VRE cases should be interpreted with care. Case–control studies [4,11-14,16,17,19,25] comparing VRE to VSE bacteraemia patients may not accurately account for the impact of vancomycin use; this bias is minimised with a case-case-control study that compares VRE bacteraemia patients to controls without enterococcal bacteraemia and VSE bacteraemia patients to controls [33]. Of the two studies involving predominantly *vanB* genotype [30,31], only one [31] was a case-case-control study which used the same control patients for both case groups, thereby enabling meaningful characterisation of the distinct features of bacteraemia specific to the resistant and susceptible enterococci [33]. However, the external validity of the results from this single centre study is limited [31]. Furthermore, that study [31] and another involving *vanA* VRE [12] do not adjust for patient co-morbidities, and duration of hypoalbuminaemia and neutropenia. Other studies [4,9-11,13-30] did not adjust for a combination of factors (co-morbidities and/or duration of antibiotic therapy, hypoalbuminaemia, and/or neutropenia).

Studies have found that neutropenia, use of antibiotics (e.g. cephalosporins, imipenem and metronidazole), urinary catheter and central venous catheter use, older age, gastrointestinal disease, biliary complications, severe mucositis, a higher severity of illness score and low plasma albumin levels were linked to VSE bacteraemia [31,34-40]. Unfortunately, the majority of these studies on factors linked to VSE bacteraemia were descriptive (had small sample size) [34-37], and did not adjust for duration of antibiotic therapy, hypoalbuminaemia and/or neutropenia [31,38,39].

Given that enterococcal bacteraemia is increasing and data on factors linked to *vanB* VRE bacteraemia remain limited, an understanding of the factors associated with *vanB* VRE and VSE bacteraemia would facilitate efforts to prevent and manage these infections. Thus, the primary aim of this study was to identify the potential factors associated with *vanB* VRE bacteraemia and secondarily, to compare them with those for VSE bacteraemia.

## Methods

A retrospective case-case-control study was conducted at two tertiary public hospitals, The Alfred (~420 acute-care beds) and Austin Health (~400 acute-care beds) in Victoria, Australia. Both institutions service adult patients, have intensive care units and emergency departments, infectious diseases and infection control units, haematology-oncology services and provide other specialist services. This study was approved by the Human Research Ethics Committees of both hospitals and Monash University. Waiver of patient consent was approved by all ethics committees because no patient recruitment of follow-up occurred, and data utilised in this study included information already collected as part of patient care.

All study participants were admitted to the hospital between January 2002 and March 2010 (inclusive). The sample size was based around all cases of VRE bacteraemia identified during the study period. The study period was specified a priori, from January 2002 to March 2010, as the infection control interventions against VRE in both hospitals did not change substantially over this time period. Only patients with hospital length of stay (LOS) > 2 days were eligible for inclusion in the study. Pregnant patients and those < 18 years of age were excluded. Selection of all patients was made without knowledge of patient outcomes. Each patient was only included once. Patients from whom one or more blood cultures were positive for VRE were classified as VRE cases. The VRE genotype was determined by polymerase chain reaction [5,41]. Patients who had one or more blood cultures positive for VSE were also identified (VSE cases). Controls without evidence of enterococcal bacteraemia were identified from a list of patients admitted to the hospital and may have had infections (including bacteraemia) due to other pathogens. Matching was performed within the same hospital on a 1:1:1 basis. VSE cases and controls were matched with VRE cases according to date of admission (within 2 years of VRE case admission date), and where possible, unit of admission. Where more than one VSE case or control was eligible for matching on the same admission date, the VSE case or control was randomly chosen.

The present analysis included additional data (e.g. patient co-morbidities, and duration of hypoalbuminaemia and neutropenia) which were not considered in the study discussed above [31] and patients from another hospital. Information on patient demographics and clinical characteristics, antimicrobial use, and outcomes of hospitalisation were collected via a retrospective review of patients’ medical records by the same researcher (ALYC). These criteria for the exposure and outcome measures were pre-determined prior to data collection.

Polymicrobial bacteraemia was the isolation of one or more bacterial or fungal pathogens within 24 hours from the same or different blood sample where the initial VRE or VSE was isolated [17]. Chronic Disease Score specific to VRE (CDS-VRE), which has been validated for use in studies on factors linked to disease, was used to measure patient co-morbidities [42]. Gastrointestinal disease included a history of liver disease, peptic ulcers, diverticulitis, graft-versus-host-disease of the gut, inflammatory bowel disease, colon cancer and colorectal cancer. Neutropenia, hypoalbuminaemia, exposure to medical devices, and antibiotic-specific days were defined, respectively, as number of days where neutrophils were < 500/mm^3^, serum or plasma albumin < 35 g/L, exposure to central lines, mechanical ventilation, urinary catheter and total parenteral nutrition, and total number of days that antibiotic(s) were administered orally or intravenously, within 30 days prior to bacteraemia (for VRE and VSE cases) or death or discharge (controls).

Data analyses were performed with Stata, version 12.0 (Stata Corporation, College Station, Texas, USA). Categorical variables are expressed as frequencies and percentages. For continuous variables, mean or median are reported for normally or non-normally distributed data, respectively. Conditional logistic regression was used to assess the association between each independent variable and the presence or absence of VRE or VSE bacteraemia. We assessed the assumption of a linear relationship between log odds and each of the continuous exposure factors in the multivariable models for VRE and VSE bacteraemia. The continuous exposure factors were analysed as continuous variables with coefficients expressed as odds ratio because the relationships between log odds and each of the continuous exposure factors were approximately linear. Variables that were significant on univariable analysis, or biologically plausible factors linked to disease, or potential confounders, were inserted into the multivariable models. Link test was used to assess the fit of each of the multivariable models for VRE and VSE bacteraemia [43]. All statistical tests were two-tailed and a p < 0.05 was considered significant.

## Results

Of the 724 patients with enterococcal bacteraemia identified during the study period, 121 (17%) and 603 (83%) were VRE and VSE cases, respectively. A final number of 116 VRE cases (and corresponding matched VSE cases and controls) were reviewed given four VRE patients had missing medical records and one VRE patient was pregnant. The ratio of enterococcal isolates that were vancomycin-resistant to -susceptible was 6% in 2002, and increased to 39% in 2009 and 22% in the first quarter of 2010. All VRE isolates were *vanB* genotype.

Demographics of the VRE and VSE cases, and controls are shown in Table 1. For the studied admission, the reason for admission and co-morbidities were similar between cases and controls. Most VRE isolates were *Enterococcus faecium*, whereas the VSE isolates were predominantly *E. faecalis.* To account for this difference, factors associated with VSE bacteraemia due to *E. faecium* (haematological malignancy and duration of neutropenia) and *E. faecalis* (urinary catheter use) [44], were included in the multivariable analysis. We also accounted for time at risk for enterococcal bacteraemia by adjusting in the multivariable analyses for the following factors occurring in the 30 days prior to bacteraemia (for VRE and VSE cases) and death or discharge (for controls): number of days of antibiotic administration, neutropenia and hypoalbuminaemia, and use of medical devices (e.g. catheters). Adjustment for age was performed on multivariable analysis when age was found to be significant on univariable analysis.

**Table 1:** Patient demographics

**Characteristics**	**VRE cases (n = 116)**	**VSE cases (n = 116)**	**Controls (n = 116)**
Age, median (IQR), years	60 (47–68)	63.5 (51–76)	58 (44–68)
Sex			
Male	69 (59)	72 (62)	63 (54)
Female	47 (41)	44 (38)	53 (46)
Unit of admission			
Haematology/oncology	61 (53)	43 (37)	67 (58)
Non-haematology/oncology	55 (47)	73 (63)	49 (42)
Reason for admission			
Medical	89 (77)	87 (75)	89 (77)
Surgical	27 (23)	29 (25)	27 (23)
Charlson score, median (IQR)	4 (2–5)	3 (1–5)	4 (1–4)
Chronic disease score-VRE (CDS-VRE), median (IQR)	1 (0–1.9)	0 (0–1)	0 (0–1.6)
Lung disease	17 (15)	17 (15)	17 (15)
Ischaemic heart disease	25 (22)	23 (20)	11 (9)
Myocardial infarction	10 (9)	10 (9)	3 (3)
Congestive heart failure	13 (11)	14 (12)	2 (2)
Cerebrovascular disease	7 (6)	13 (11)	5 (4)
Gastrointestinal disease	54 (47)	64 (55)	38 (33)
Renal disease	21 (18)	18 (16)	15 (13)
Diabetes mellitus	25 (22)	30 (26)	20 (17)
Cancer	74 (64)	64 (55)	74 (64)
Solid organ transplant recipient	14 (12)	7 (6)	5 (4)
Enterococcus species			
*E. faecalis*	9 (8)	71 (61)	-
*E. faecium*	107 (92)	39 (34)	-
*E. casseliflavus, E. gallinarum,* or *E. durans*	-	4 (3)	-
Both *E. faecalis* and *E. faecium*	-	1 (1)	-
Unknown	-	1 (1)	-
Polymicrobial bacteraemia	33 (28)	50 (43)	-
Infection (other than due to Enterococci) prior to bacteraemia (VRE and VSE cases) and prior to discharge (controls)	61 (53)	43 (37)	34 (29)
Days from admission until bacteraemia, median (IQR), days	16 (7–24)	7.5 (1–18.5)	-
Total length of stay, median (IQR), days	35 (22.5-50.5)	25 (16–45.5)	9 (5–20)
In-hospital mortality	42 (36)	30 (26)	5 (4)

Note:

Data are number (%) of patients unless indicated otherwise.

Percentages were rounded to the nearest whole number.

In patients with *vanB* VRE bacteraemia, 54 (47%), 22 (19%) and 14 (12%) were treated with teicoplanin monotherapy, linezolid monotherapy and no antibiotics, respectively. For 26 (22%) *vanB* VRE bacteraemia patients, a combination (concurrent or in sequence) of teicoplanin, linezolid, quinupristin-dalfopristin or benzylpenicillin was administered as definitive therapy. In VSE patients, 46 (40%), 21 (18%), 1 (1%), 9 (8%), 6 (5%), and 2 (2%) patients were administered definitive therapy using intravenous glycopeptides (teicoplanin or vancomycin), penicillins (ampicillin, benzylpenicillin, ticarcillin-clavulanic acid, piperacillin-tazobactam), meropenem, no antibiotics, combination of intravenous gentamicin and ampicillin, and oral linezolid, respectively. In 30 (26%) VSE patients, a combination (concurrent or in sequence) of vancomycin, teicoplanin, linezolid, ampicillin, benzylpenicillin or meropenem was administered.

Tables 2 and 3 summarise the analyses of factors linked to VRE and VSE bacteraemia, respectively. In the multivariable analysis, a higher CDS-VRE score, an increase in duration of metronidazole therapy, and use of urinary catheters within the previous 30 days were associated with increased odds of *vanB* VRE bacteraemia (Table 2). When patients with VSE bacteraemia were compared to controls (Table 3), after adjustment for confounders, admission to the haematology/oncology unit (compared to non-haematology/oncology units) was associated with lower odds of VSE bacteraemia whilst, gastrointestinal disease and duration of metronidazole use were associated with higher odds of VSE bacteraemia.

**Table 2:** **Factors associated with *vanB *** **VRE bacteraemia**

**Factors associated with *vanB *** **VRE bacteraemia**	**VRE cases (n = 116)**	**Controls (n = 116)**	**Univariable**			**Multivariable**		
			**OR**	**95% CI**	**p-value**	**OR**	**95% CI**	**p-value**
Age, median (IQR), years	60 (47–68)	58 (44–68)	1.01	0.99-1.02	0.481			
Female	47 (41)	53 (46)	0.81	0.48-1.36	0.432			
Transfer from another hospital	28 (24)	14 (12)	2.56	1.18-5.52	0.017			
Unit of admission								
Non-haematology/oncology to read Non-haematology/oncology	55 (47)	49 (42)	Reference					
Haematology/ oncology	61 (53)	67 (58)	0	-	-			
ICU admission in prior 30 days	39 (34)	13 (11)	6.57	2.97-14.55	<0.001			
CDS-VRE score, median (IQR)	1 (0–1.9)	0 (0–1.6)	1.36	1.02-1.80	0.036	1.70	1.05-2.77	0.032
*Clostridium difficile* toxin positive	5 (4)	1 (1)	5.00	0.58-42.80	0.142			
Infection(s) due to pathogens other than Enterococci	61 (53)	34 (29)	2.93	1.60-5.37	0.001	0.97	0.39-2.46	0.953
Gastrointestinal disease	54 (47)	38 (33)	2.00	1.10-3.64	0.024			
Liver disease	16 (14)	15 (13)	1.14	0.41-3.15	0.796			
Haematological malignancy	66 (57)	63 (54)	1.6	0.52-4.89	0.410	0.41	0.06-2.61	0.342
Bone marrow transplantation type								
Nil	97 (84)	100 (86)	Reference			Reference		
Autologous	3 (3)	7 (6)	0.33	0.07-1.65	0.178	0.27	0.03-2.22	0.221
Allogeneic	16 (14)	9 (8)	2.40	0.85-6.81	0.100	2.79	0.57-13.66	0.206
Ceftriaxone days, median (IQR), days	0 (0)	0 (0)	1.09	0.98-1.22	0.130	1.16	0.91-1.48	0.234
Third generation cephalosporin days , median (IQR), days^a^	0 (0–1)	0 (0–0.49)	1.04	0.95-1.13	0.439			
Fluoroquinolone days, median (IQR), days^b^	1 (0–7.63)	0 (0–2.42)	1.07	1.01-1.12	0.016			
Metronidazole days, median (IQR), days	0 (0–1.78)	0 (0)	1.54	1.16-2.04	0.003	1.65	1.17-2.33	0.004
Ticarcillin-clavulanic acid days, median (IQR), days	0 (0)	0 (0)	1.02	0.92-1.13	0.674			
Piperacillin-tazobactam days, median (IQR), days	0 (0–2.18)	0 (0)	1.04	0.96-1.14	0.352			
Meropenem days, median (IQR), days	0 (0–5.94)	0 (0)	1.15	1.06-1.25	0.001			
Vancomycin days, median (IQR), days	2.58 (0–8)	0 (0–2.18)	1.10	1.04-1.17	0.002	1.04	0.95-1.15	0.401
Neutropenia days, median (IQR), days	1 (0–10)	0 (0–1)	1.08	1.03-1.14	0.003	1.04	0.96-1.11	0.341
Hypoalbuminaemia days, median (IQR), days	13 (7.5-20)	5.5 (2–14.5)	1.08	1.04-1.12	<0.001	0.97	0.91-1.04	0.435
Central line use	94 (81)	55 (47)	5.22	2.56-10.66	<0.001	3.06	0.94-9.99	0.063
Mechanical ventilation	29 (25)	7 (6)	5.40	2.08-14.02	0.001			
Urinary catheter	57 (49)	27 (23)	3.50	1.84-6.65	<0.001	2.86	1.09-7.53	0.033
Parenteral nutrition	28 (24)	8 (7)	3.86	1.68-8.86	0.001			

Note:

Data are number (%) of patients unless indicated otherwise.

^a^Third generation cephalosporins include cefotaxime, ceftriaxone, and ceftazidime.

^b^Fluoroquinolones include moxifloxacin, norfloxacin and ciprofloxacin.

**Table 3:** Factors associated with VSE bacteraemia

**Factors associated with VSE bacteraemia**	**VSE cases (n = 116)**	**Controls (n = 116)**	**Univariable**			**Multivariable**		
			**OR**	**95% CI**	**p-value**	**OR**	**95% CI**	**p-value**
Age, median (IQR), years	63.5 (51–76)	58 (44–68)	1.02	1.00-1.04	0.019	1.01	0.99-1.03	0.305
Female	44 (38)	53 (46)	0.74	0.44-1.23	0.243			
Transfer from another hospital	21 (18)	14 (12)	1.64	0.77-3.46	0.198			
Unit of admission								
Non-haematology/oncology to read Non-haematology/oncology	73 (63)	49 (42)	Reference			Reference		
Haematology/oncology	43 (37)	67 (58)	0.04	0.01-0.30	0.002	0.08	0.01-0.74	0.026
ICU admission in prior 30 days	29 (25)	13 (11)	2.60	1.25-5.39	0.010	1.71	0.52-5.58	0.373
CDS-VRE score, median (IQR)	0 (0–1)	0 (0–1.6)	0.89	0.66-1.20	0.446			
*Clostridium difficile* toxin positive	2 (2)	1 (1)	2.00	0.18-22.06	0.571			
Infection(s) due to pathogens other than Enterococci	43 (37)	34 (29)	1.45	0.82-2.56	0.201			
Gastrointestinal disease	64 (55)	38 (33)	3.00	1.60-5.62	0.001	2.29	1.05-4.99	0.037
Liver disease	13 (11)	15 (13)	0.71	0.23-2.25	0.566			
Haematological malignancy	35 (30)	63 (54)	0.15	0.06-0.39	<0.001	0.40	0.12-1.33	0.134
Bone marrow transplantation type								
Nil	105 (91)	100 (86)		Reference			Reference	
Autologous	2 (2)	7 (6)	0.29	0.06-1.38	0.118			
Allogeneic	9 (8)	9 (8)	1.00	0.38-2.66	1.000			
Ceftriaxone days, median (IQR), days	0 (0–0.5)	0 (0)	1.10	0.99-1.24	0.088			
Third generation cephalosporin days , median (IQR), days^a^	0 (0–1)	0 (0–0.49)	1.03	0.94-1.13	0.523			
Fluoroquinolone days, median (IQR), days^b^	0 (0–1.04)	0 (0–2.42)	0.96	0.91-1.02	0.190			
Metronidazole days, median (IQR), days	0 (0)	0 (0)	1.23	1.06-1.43	0.007	1.23	1.02-1.48	0.032
Ticarcillin-clavulanic acid days, median (IQR), days	0 (0)	0 (0)	1.02	0.93-1.11	0.711			
Piperacillin-tazobactam days, median (IQR), days	0 (0)	0 (0)	0.89	0.79-1.00	0.054			
Meropenem days, median (IQR), days	0 (0)	0 (0)	1.00	0.92-1.08	0.934			
Vancomycin days, median (IQR), days	0 (0–1.25)	0 (0–2.18)	0.97	0.92-1.03	0.343			
Neutropenia days, median (IQR), days	0	0 (0–1)	0.98	0.94-1.02	0.344	1.00	0.94-1.06	0.911
Hypoalbuminaemia days, median (IQR), days	7 (1–16)	5 (1–13.5)	1.01	0.98-1.05	0.479	1.00	0.95-1.05	0.888
Central line use	59 (51)	55 (47)	1.17	0.67-2.05	0.572			
Mechanical ventilation	17 (15)	7 (6)	2.67	1.04-6.81	0.040			
Urinary catheter	46 (40)	27 (23)	2.12	1.19-3.77	0.011	1.16	0.47-2.88	0.741
Parenteral nutrition	16 (14)	8 (7)	2.00	0.86-4.67	0.109			

Note:

Data are number (%) of patients unless indicated otherwise.

^a^Third generation cephalosporins include cefotaxime, ceftriaxone, and ceftazidime.

^b^Fluoroquinolones include moxifloxacin, norfloxacin and ciprofloxacin.

## Discussion and conclusions

Unlike earlier studies [4,23,25-27], the current study only included data from patients who had *vanB* VRE bacteraemia and did not assess colonised patients. It is not a single-centre study [4,9-12,15-27,30,31] and, to our knowledge, is the largest case-case-control study involving *vanB* VRE bacteraemia. The present report is the only case-case-control study to identify the factors associated with *vanB* VRE and VSE bacteraemia, while adjusting for patient co-morbidities and duration of antibiotic therapy, hypoalbuminaemia, and neutropenia*.*

The association between patient co-morbidities, as measured by the CDS-VRE score, and development of *vanB* VRE bacteraemia was investigated. The CDS-VRE score was calculated based on medications ordered within 24 hours of hospital admission for medical conditions such as diabetes, peptic ulcers, kidney disease, history of transplantation and cancer [42]. A weight is assigned for each medical condition and the final CDS-VRE score is a weighted sum of the medical conditions for each patient. The presence of a higher number of co-morbidities included in the score is associated with a higher CDS-VRE score. Co-morbidities related to immune-suppression such as transplantation and cancer were given a higher weighting, compared to diabetes and peptic ulcer. As such, patients with co-morbidities associated with immune-suppression tend to have high CDS-VRE scores. Our results show that patients with a higher CDS-VRE score have higher odds for *vanB* VRE bacteraemia. This was consistent with studies that reported association between haematological malignancy and *vanA* VRE bacteraemia [11]. Diabetes [20], acute renal failure [19], and severity of gut mucositis [18] may predispose patients to the development of VRE bacteraemia; VRE genotype was not determined in these studies.

Antibiotic use has been linked to VRE colonisation in the gastrointestinal tract and/or infection, including bacteraemia [19,26,45]. In the present study, duration of therapy with specific antibiotics was considered, unlike many earlier studies [12,13,16,17,19-22,24,27,28,30]. Exposure to anti-anaerobic antibiotics has been linked to VRE gastrointestinal tract colonisation [45] and bacteraemia [10,46]; however, the VRE genotype was either *vanA* VRE [10,46] or not determined [45]. In our study, an increase in duration of therapy with metronidazole (an anti-anaerobic antibiotic) was linked to VRE bacteraemia. Whilst exposure to metronidazole may be a marker for bacteraemia with suspected intra-abdominal source, this was not explored as it was beyond the scope of the current work. A recent study reported an association between increased ceftriaxone usage and VRE bacteraemia (genotype not reported) [29]. An association with ceftriaxone therapy, however, was not observed in our study which adjusted for patient co-morbidities. Although VRE overgrowth and subsequent VRE colonisation and/or infection may occur with vancomycin exposure [11,13], multivariable analysis suggests that the use of vancomycin was not associated with increased odds of VRE bacteraemia in this study. This is probably due to a reduction in bias [33,47] as the present study did not use VSE as controls, in contrast to studies that had included VSE patients as controls [11,13]. In this study, the controls without enterococcal bacteraemia have had exposure to vancomycin. Thus, a comparison of VRE to VSE bacteraemia patients could be made to identify factors linked to VRE bacteraemia; however, we chose to compare VRE bacteraemia patients to controls without enterococcal bacteraemia in a case-case–control study as it will minimise the selection bias that affects the identification, and the magnitude, of the effect due to vancomycin [33,47]. To further minimise selection bias related to choice of controls in the current study, VSE patients and controls were randomly chosen and matched to VRE cases for time of admission and wherever possible, unit of admission.

Similar to studies that demonstrated VRE bacteraemia was associated with exposure of patients to VRE contaminated ‘exogenous’ sources [16,31], urinary catheter use was independently associated with the development of VRE bacteraemia. The need for a urinary catheter may also be an indicator of severe underlying illness [16]. Compared to an earlier study that only considered hypoalbuminaemia and neutropenia in a binary manner (i.e. presence or absence) [31], in the present study the durations of these conditions were found to be linked to VRE bacteraemia. The use of a continuous variable (i.e. duration) provides a more robust measure of exposure to these potential factors linked to disease.

The duration of metronidazole therapy was linked to VSE bacteraemia, similar to our finding for *vanB* VRE bacteraemia. However, other factors linked to disease differed between these two types of bacteraemia. We found that a history of gastrointestinal disease was associated with VSE bacteraemia. Changes in the gastrointestinal tract may predispose to the migration of gut micro-organisms such as enterococci, increasing the link to VSE bacteraemia [48]. Due to the difficulty in matching VSE cases to VRE cases from the haematology/oncology unit, we adjusted for unit of admission in the multivariable analysis for VSE bacteraemia. Interestingly, admission to the haematology/oncology unit compared to other units was associated with reduced odds of VSE bacteraemia. This finding may be due to an unmeasured confounder such as exposure in this group of patients to antibiotics that are active against VSE, thereby reducing the odds of VSE bacteraemia. Unlike earlier studies that did not adjust for duration of hypoalbuminaemia and neutropenia [31,38], the present study found no association between the duration of low albumin and neutrophil levels, and odds of VSE bacteraemia.

Through this case-case–control study we were able to differentiate the factors associated with VRE and VSE bacteraemia. It is recognised that data collected retrospectively may be subject to variability in reporting from different clinicians and missing data. Whilst the effect of individual enterococcal species on the results of this study was not specifically studied given the sample size, the potential effects of enterococcal species on study findings were accounted for in the analysis. Failure to adjust for confounding may result in falsely elevated or reduced odds ratio [49]. Accordingly, multivariable analyses were also performed in the current study to minimise bias associated with confounding.

This study, the largest published case-case–control study involving only *vanB* VRE bacteraemia has identified and differentiated the factors associated with *vanB* VRE and VSE bacteraemia. VSE bacteraemia was linked to a history of gastrointestinal disease. In contrast, a higher burden of patient co-morbidities and urinary catheter use were associated with *vanB* VRE bacteraemia.

## Competing interest

DCMK has sat on advisory board for Merck, Sharp & Dohme, and Pfizer. He receives financial support (not related to the current work) from Pfizer, Novartis, Merck and Gilead Sciences. All other authors: No relevant disclosures.

## Authors’ contributions

ALYC was involved in the design of the study, carried out the data collection, performed the statistical analyses, drafted the manuscript. TP, BN, DS, MLG was involved in the design of the study and critical review of the manuscript, and advised on data collection. RN and DK participated in the design of the study, advised on data collection, and helped to draft the manuscript. All authors read and approved the final manuscript.

## Pre-publication history

The pre-publication history for this paper can be accessed here:

http://www.biomedcentral.com/1471-2334/14/353/prepub

## References

[B1] Hidron Alicia I Edwards Jonathan R Patel J Horan Teresa C Sievert Dawn M Pollock Daniel A Fridkin Scott K NHSN annual update: antimicrobial-resistant pathogens associated with healthcare-associated infections: annual summary of data reported to the national healthcare safety network at the centers for disease control and prevention, 2006–2007 Infect Control Hosp Epidemiol 2008 14 996 1011 10.1086/591861 18947320

[B2] Deshpande LM Fritsche TR Moet GJ Biedenbach DJ Jones RN Antimicrobial resistance and molecular epidemiology of vancomycin-resistant *Enterococci* from North America and Europe: a report from the SENTRY antimicrobial surveillance program Diagn Microbiol Infect Dis 2007 14 163 170 10.1016/j.diagmicrobio.2006.12.022 17368801

[B3] Australian Group for Antimicrobial Resistance (AGAR) posters and publications website. Vancomycin resistant enterococci in Australia: results of the AGAR surveys 1995 to 2010 http://www.agargroup.org/files/VRE%20in%20Australia.pdf. Accessed 7 June, 2012

[B4] Webb M Riley L Roberts R Cost of hospitalization for and risk factors associated with vancomycin-resistant *Enterococcus faecium* infection and colonization Clin Infect Dis 2001 14 445 452 10.1086/321891 11462178

[B5] Bell JM Paton JC Turnidge J Emergence of vancomycin-resistant *Enterococci* in Australia: phenotypic and genotypic characteristics of isolates J Clin Microbiol 1998 14 2187 2190 9665988 10.1128/jcm.36.8.2187-2190.1998 PMC105003

[B6] Schouten MA Hoogkamp-Korstanje JA Meis JF Voss A Prevalence of vancomycin-resistant enterococci in Europe Eur J Clin Microbiol Infect Dis 2000 14 816 822 10.1007/s100960000390 11152305

[B7] Stuart RL Kotsanas D Webb B Vandergraaf S Gillespie EE Hogg GG Korman TM Prevalence of antimicrobial-resistant organisms in residential aged care facilities Med J Aust 2011 14 530 533 10.5694/mja11.10724 22060088

[B8] Burrell LJ Grabsch EA Padiglione AA Grayson ML Prevalence of colonisation with vancomycin-resistant enterococci (VRE) among haemodialysis outpatients in Victoria: implications for screening Med J Aust 2005 14 492 15865598 10.5694/j.1326-5377.2005.tb06794.x

[B9] Montecalvo MA Horowitz H Gedris C Carbonaro C Tenover FC Issah A Cook P Wormser GP Outbreak of vancomycin-, ampicillin-, and aminoglycoside-resistant *Enterococcus faecium* bacteremia in an adult oncology unit Antimicrob Agents Chemother 1994 14 1363 1367 10.1128/AAC.38.6.1363 8092838 PMC188211

[B10] Edmond MB Ober JF Weinbaum DL Pfaller MA Hwang T Sanford MD Wenzel RP Vancomycin-resistant *Enterococcus faecium* bacteremia: risk factors for infection Clin Infect Dis 1995 14 1126 1133 10.1093/clinids/20.5.1126 7619987

[B11] Shay DK Maloney SA Montecalvo M Banerjee S Wormser GP Arduino MJ Bland LA Jarvis WR Epidemiology and mortality risk of vancomycin-resistant enterococcal bloodstream infections J Infect Dis 1995 14 993 1000 10.1093/infdis/172.4.993 7561221

[B12] Peset V Tallon P Sola C Sanchez E Sarrion A Perez-Belles C Vindel A Canton E Gobernado M Epidemiological, microbiological, clinical, and prognostic factors of bacteremia caused by high-level vancomycin-resistant *Enterococcus* species Eur J Clin Microbiol Infect Dis 2000 14 742 749 10.1007/s100960000360 11117637

[B13] Bhavnani SM Drake JA Forrest A Deinhart JA Jones RN Biedenbach DJ Ballow CH A nationwide, multicenter, case–control study comparing risk factors, treatment, and outcome for vancomycin-resistant and -susceptible enterococcal bacteremia Diagn Microbiol Infect Dis 2000 14 145 158 10.1016/S0732-8893(99)00136-4 10729656

[B14] Vergis EN Hayden MK Chow JW Snydman DR Zervos MJ Linden PK Wagener MM Schmitt B Muder RR Determinants of vancomycin resistance and mortality rates in enterococcal bacteremia: a prospective multicenter study Ann Intern Med 2001 14 7 484 492 10.7326/0003-4819-135-7-200110020-00007 11578151

[B15] Roghmann M-C McCarter RJ Brewrink J Cross AS Morris JG *Clostridium difficile* infection is a risk factor for bacteremia due to vancomycin-resistant *Enterococci* (VRE) in VRE-colonized patients with acute leukemia Clin Infect Dis 1997 14 1056 1059 10.1086/516112 9402356

[B16] Stosor V Peterson LR Postelnick M Noskin GA *Enterococcus faecium* bacteremia: does vancomycin resistance make a difference? Arch Intern Med 1998 14 522 527 10.1001/archinte.158.5.522 9508230

[B17] Lucas GM Lechtzin N Puryear DW Yau LL Flexner CW Moore RD Vancomycin-resistant and vancomycin-susceptible enterococcal bacteremia: comparison of clinical features and outcomes Clin Infect Dis 1998 14 1127 1133 10.1086/520311 9597241

[B18] Kuehnert MJ Jernigan JA Pullen AL Rimland D Jarvis WR Association between mucositis severity and vancomycin-resistant enterococcal bloodstream infection in hospitalized cancer patients Infect Control Hosp Epidemiol 1999 14 660 663 10.1086/501561 10530642

[B19] Lautenbach E Bilker WB Brennan PJ Enterococcal bacteremia: risk factors for vancomycin resistance and predictors of mortality Infect Control Hosp Epidemiol 1999 14 318 323 10.1086/501624 10349947

[B20] Zaas A Song X Tucker P Perl T Risk factors for development of vancomycin-resistant enterococcal bloodstream infection in patients with cancer who are colonized with vancomycin-resistant *Enterococci* Clin Infect Dis 2002 14 1139 1146 10.1086/342904 12410472

[B21] de Perio MA Yarnold Paul R Warren J Noskin Gary A Risk factors and outcomes associated with non–*Enterococcus faecalis*, non–*Enterococcus faecium* enterococcal bacteremia Infect Contr Hosp Epidemiol 2006 14 28 33 10.1086/500000 16418983

[B22] Olivier C Blake R Steed L Salgado C Risk of vancomycin-resistant *Enterococcus* (VRE) bloodstream infection among patients colonized with VRE Infect Control Hosp Epidemiol 2008 14 404 409 10.1086/587647 18419361

[B23] Morris JG Shay DK Hebden JN McCarter RJ Perdue BE Jarvis W Johnson JA Dowling TC Polish LB Schwalbe RS *Enterococci* resistant to multiple antimicrobial agents, including vancomycin: establishment of endemicity in a university medical center Ann Intern Med 1995 14 250 259 10.7326/0003-4819-123-4-199508150-00002 7611590

[B24] Papanicolaou G Meyers B Meyers J Mendelson M Lou W Emre S Sheiner P Miller C Nosocomial infections with vancomycin-resistant *Enterococcus faecium* in liver transplant recipients: risk factors for acquisition and mortality Clin Infect Dis 1996 14 760 766 10.1093/clinids/23.4.760 8909841

[B25] Tornieporth NG Roberts RB John J Hafner A Riley LW Risk factors associated with vancomycin-resistant *Enterococcus faecium* infection or colonization in 145 matched case patients and control patients Clin Infect Dis 1996 14 767 772 10.1093/clinids/23.4.767 8909842

[B26] Carmeli Y Eliopoulos GM Samore MH Antecedent treatment with different antibiotic agents as a risk factor for vancomycin-resistant *Enterococcus* Emerg Infect Dis 2002 14 802 807 10.3201/eid0808.010418 12141965 PMC2732508

[B27] Chavers LS Moser SA Funkhouser E Benjamin WH Jr Chavers P Stamm AM Waites KB Association between antecedent intravenous antimicrobial exposure and isolation of vancomycin-resistant *Enterococci* Microb Drug Resist 2003 14 S69 S77 10.1089/107662903322541928 14633370

[B28] Nguyen GC Leung W Weizman AV Increased risk of vancomycin-resistant *Enterococcus* (VRE) infection among patients hospitalized for inflammatory bowel disease in the United States Inflamm Bowel Dis 2011 14 1338 1342 10.1002/ibd.21519 21560197

[B29] McKinnell JA Kunz DF Chamot E Patel M Shirley RM Moser SA Baddley JW Pappas PG Miller LG Association between vancomycin-resistant *Enterococci* bacteremia and ceftriaxone usage Infect Control Hosp Epidemiol 2012 14 718 724 10.1086/666331 22669234 PMC3879097

[B30] Worth LJ Thursky KA Seymour JF Slavin MA Vancomycin-resistant *Enterococcus faecium* infection in patients with hematologic malignancy: patients with acute myeloid leukemia are at high-risk Eur J Haematol 2007 14 226 233 10.1111/j.1600-0609.2007.00911.x 17655696

[B31] Peel T Cheng AC Spelman T Huysmans M Spelman D Differing risk factors for vancomycin-resistant and vancomycin-sensitive enterococcal bacteraemia Clin Microbiol Infect 2011 14 388 394 21848977 10.1111/j.1469-0691.2011.03591.x

[B32] Patel R Clinical impact of vancomycin-resistant *Enterococci* J Antimicrob Chemother 2003 14 iii 13 21 10.1093/jac/dkg272 12801938

[B33] Kaye KS Harris AD Samore M Carmeli Y The case-case–control study design: addressing the limitations of risk factor studies for antimicrobial resistance Infect Contr Hosp Epidemiol 2005 14 346 351 10.1086/502550 15865269

[B34] Barrall DT Kenney PR Slotman GJ Burchard KW Enterococcal bacteremia in surgical patients Arch Surg 1985 14 57 63 10.1001/archsurg.1985.01390250049008 3966874

[B35] Pallares R Pujol M Pena C Ariza J Martin R Gudiol F Cephalosporins as risk factor for nosocomial *Enterococcus faecalis* bacteremia: a matched case–control study Arch Intern Med 1993 14 1581 1586 10.1001/archinte.1993.00410130103010 8323421

[B36] Mikulska M Del Bono V Prinapori R Boni L Raiola A Gualandi F Van Lint M Dominietto A Lamparelli T Cappellano P Bacigalupo A Viscoli C Risk factors for enterococcal bacteremia in allogeneic hematopoietic stem cell transplant recipients Transpl Infect Dis 2010 14 505 512 10.1111/j.1399-3062.2010.00544.x 20636482

[B37] Gray J Marsh PJ Stewart D Pedler SJ Enterococcal bacteraemia: a prospective study of 125 episodes J Hosp Infect 1994 14 179 186 10.1016/0195-6701(94)90125-2 7963458

[B38] Caballero-Granado FJ Becerril B Cisneros JM Cuberos L Moreno I Pachon J Case–control study of risk factors for the development of enterococcal bacteremia Eur J Clin Microbiol Infect Dis 2001 14 83 90 11305477 10.1007/s100960000429

[B39] Sitges-Serra A Lopez M Girvent M Almirall S Sancho J Postoperative enterococcal infection after treatment of complicated intra-abdominal sepsis Br J Surg 2002 14 361 367 10.1046/j.0007-1323.2001.02023.x 11872065

[B40] Patel R Badley AD Larson-Keller J Harmsen WS Ilstrup DM Wiesner RH Steers JL Krom RA Portela D Cockerill FR Paya CV Relevance and risk factors of enterococcal bacteremia following liver transplantation Transplantation 1996 14 1192 1197 10.1097/00007890-199604270-00013 8610417

[B41] Dutka-Malen S Evers S Courvalin P Detection of glycopeptide resistance genotypes and identification to the species level of clinically relevant enterococci by PCR J Clin Microbiol 1995 14 24 27 7699051 10.1128/jcm.33.1.24-27.1995 PMC227872

[B42] McGregor JC Perencevich EN Furuno JP Langenberg P Flannery K Zhu J Fink JC Bradham DD Harris AD Comorbidity risk-adjustment measures were developed and validated for studies of antibiotic-resistant infections J Clin Epidemiol 2006 14 1266 1273 10.1016/j.jclinepi.2006.01.016 17098569

[B43] Pregibon D Goodness of link tests for generalized linear models J Roy Stat Soc C Appl Stat 1980 14 15 24

[B44] Suppola JP Kuikka A Vaara M Valtonen VV Comparison of risk factors and outcome in patients with *Enterococcus faecalis* vs *Enterococcus faecium* bacteraemia Scand J Infect Dis 1998 14 153 157 10.1080/003655498750003546 9730302

[B45] Donskey CJ Chowdhry TK Hecker MT Hoyen CK Hanrahan JA Hujer AM Hutton-Thomas RA Whalen CC Bonomo RA Rice LB Effect of antibiotic therapy on the density of vancomycin-resistant *Enterococci* in the stool of colonized patients New Engl J Med 2000 14 1925 1932 10.1056/NEJM200012283432604 11136263 PMC4370337

[B46] Taur Y Xavier JB Lipuma L Ubeda C Goldberg J Gobourne A Lee YJ Dubin KA Socci ND Viale A Perales MA Jenq RR van den Brink MR Pamer EG Intestinal domination and the risk of bacteremia in patients undergoing allogeneic hematopoietic stem cell transplantation Clin Infect Dis 2012 14 905 914 10.1093/cid/cis580 22718773 PMC3657523

[B47] Harris A Samore M Lipsitch M Kaye K Perencevich E Carmeli Y Control-group selection importance in studies of antimicrobial resistance: examples applied to *Pseudomonas aeruginosa, Enterococci,* and *Escherichia coli* Clin Infect Dis 2002 14 1558 1563 10.1086/340533 12032889

[B48] Timmers GJ van der Zwet WC Simoons-Smit IM Savelkoul PH Meester HH Vandenbroucke-Grauls CM Huijgens PC Outbreak of vancomycin-resistant *Enterococcus faecium* in a haematology unit: risk factor assessment and successful control of the epidemic Br J Haematol 2002 14 826 833 10.1046/j.0007-1048.2002.03339.x 11886387

[B49] Sedgwick P Case–control studies: sources of bias BMJ 2011 14 d6284 10.1136/bmj.d6284

